# The "Will to Health."

**Published:** 1920-06-12

**Authors:** 


### The Hospital

The Workers' Newspaper of Administrative Medicine and Institutional Life, Administration, National Insurance and Health.

No. 1775, Vol. LXVIII. SATURDAY, JUNE 12, 1920. PRICE TWOPENCE

### THE "WILL TO HEALTH."

The Ministry of Health is an institution which, once firmly rooted in an educated and kindly public opinion, should grow into one of the most important branches of our democratic system of government. All students of social and political economy will recognise as a simple axiom that the greatest wealth of a nation is its health. A healthy and increasing population is the greatest asset which a nation can possess in any reconstructional endeavours. We deplore the fact that our health assets are not what they should be, but what are we doing to remedy this serious state of affairs?

The Government has instituted the Ministry of Health; but is this a sufficient weapon with which to equip ourselves for our great task? We think not, and realise that unless the "will to health" be thoroughly preached and the public thus educated, little in comparison with the great good that might be, will be accomplished. By the "will to health" we mean the establishment of a new racial characteristic, "the determination by all means possible to be healthy." It is common-place to remark that many illnesses are humanly preventable, and we would echo the words of the late King Edward VII.: "If preventable, why not prevented?": these words were uttered a decade ago, yet the same lamentable state of affairs exists with but little amelioration.

Personal health should be the individual responsibility of each citizen, and he should be taught from childhood upwards that it is wrong to be ill if such illness is avoidable. How can we further education among the masses of our people on the benefits of health? We would suggest a constant and persuasive stimulation of three great administrative bodies: The Government and politicians in general, the Municipal authorities and boards of guardians, and, last but not least, the Established Church of England and the various Nonconformist religions which exist in this country.

First, let us deal with the politicians. They are, as a rule, easy to influence individually, but very difficult to convert collectively. Once the "health vote" of constituents becomes numerically important, we shall get a definite political interest taken in health affairs, and each party will strive for the "health votes," in much the same way as they now strive for the "Nonconformist" or the "Irish" votes. We feel that the end result would justify these means, and consider it the duty of every patriotic citizen to approach his prospective Member of Parliament and influence him in this regard. Very much the same persuasion could be brought to bear on municipal elections, and once the various corporations and town councils became relatively more enthusiastic over health matters, such affairs as the local housing problems and insanitary areas would gradually be settled on the best principles.

The various religious denominations have a great opportunity before them; it is often said that "cleanliness is next to Godliness," and we are sure that the latter would not suffer if the former were more often preached from our pulpits. Last of all, we deal with literature and the press. In relation to the former we would sigh for a modern Dickens, but as there seems no sign of one, we must appeal to the all-puissant press. We realise that much good is already being done by the newspapers, but they have only tackled the question as yet in a half-hearted manner. What we want is a full-blooded press crusade, in which the present lamentable state of affairs may be made known to all and sundry. Let the nation really open its eyes to the fact that out of 2½ million men examined in 1918 only one in three attained the standard of Grade I! Let the "Captains of Industry" realise that some 14 million "work weeks" are lost annually through sickness among insured workers, an average of one week lost for each worker.

We are now entering an age when the workers in this country are reaping as the reward of their political agitation increasing wages and hours of recreation. It is amongst this class that preventable disease is rife; would it not be good policy to cater for the healthy use of these increasing leisure hours? We want our population playing football, not watching the efforts of trained professionals; but how are we to do this, unless we provide open spaces where games may be indulged in, and a healthy pride taken in his individual strength and fitness by each player?

We realise to the full that a colossal expenditure of money will be necessary to carry out a proper health scheme for these islands; but we also realise the enormous amount of money now being spent for the payment of war debt. This war against disease and depopulation must be fought out sooner or later, or else we shall inevitably become a declining Empire. But above all things we must imbue our people with the "will to health."

